# Exploring the role of interleukin-1β and interleukin-6 in the pathophysiology of obsessive-compulsive disorder

**DOI:** 10.1371/journal.pone.0306125

**Published:** 2024-06-26

**Authors:** Nisat Sarmin, A. S. M. Roknuzzaman, Rapty Sarker, Mamun-or- Rashid, Ahasanul Hasan, M. M. A. Shalahuddin Qusar, Eva Rahman Kabir, Md. Rabiul Islam, Zobaer Al Mahmud

**Affiliations:** 1 Faculty of Pharmacy, Department of Clinical Pharmacy and Pharmacology, University of Dhaka, Dhaka, Bangladesh; 2 Department of Pharmacy, University of Asia Pacific, Dhaka, Bangladesh; 3 Department of Psychiatry, Bangabandhu Sheikh Mujib Medical University, Shahabagh, Dhaka, Bangladesh; 4 School of Pharmacy, BRAC University, Dhaka, Bangladesh; Cedars-Sinai Medical Center, Maxine-Dunitz Neurosurgical Institute, UNITED STATES

## Abstract

**Background:**

Obsessive-compulsive disorder (OCD) is a highly prevalent neuropsychiatric disorder. Recently, there has been a growing interest in investigating the association between pro-inflammatory cytokines and the pathogenesis of OCD. However, studies targeting interleukin-1β (IL-1β) and interleukin-6 (IL-6) in OCD are limited. Therefore, the present study aimed to explore the potential role of pro-inflammatory cytokines IL-1β and IL-6 in the pathophysiology and development of OCD.

**Methods:**

This study recruited 58 OCD patients and 30 age-sex-matched healthy controls (HCs). A qualified psychiatrist diagnosed OCD patients and assessed HCs based on the Diagnostic and Statistical Manual for Mental Health Disorders, 5th edition (DSM-5) criteria. We measured the severity of OCD using the Yale-Brown Obsessive Compulsive Scale (Y-BOCS). Serum IL-1β and IL-6 levels were measured using ELISA kits following the appropriate methods.

**Results:**

The results showed that serum IL-1β levels were significantly elevated in OCD patients compared to HCs (23.68±1.65 pg/ml vs. 15.75±1.02 pg/ml; p = 0.002). Similarly, OCD patients exhibited significantly higher serum IL-6 levels than HCs (44.97±0.73 pg/ml vs. 37.04±0.35 pg/ml; p<0.001). We observed both cytokines were positively correlated with the Y-BOCS scores in OCD patients (IL-1β: r = 0.380, p = 0.015; IL-6: r = 0.324, p = 0.026) which indicates their role in disease pathophysiology.

**Conclusion:**

These results suggest that serum IL-1β and IL-6 levels may be associated with the pathophysiology of OCD. Also, these cytokines levels in blood samples can serve as early risk assessment tools for the development of OCD. We recommend further studies in a large and homogeneous population to support these findings.

## 1. Introduction

Obsessive-compulsive disorder (OCD) is a highly disabling neuropsychiatric disorder with a prevalence rate of 2–3% worldwide [1–5]. OCD is characterized by repetitive unwanted intrusive thoughts, sensations, feelings, impulses, or ideas (obsessions) that exaggerate concerns about danger, harm, or hygiene, followed by ritualized repeated behaviors or mental acts (compulsions) [2,6]. To alleviate distress from obsessions, individuals engage in compulsive behaviors. While these actions provide temporary relief, the persistent obsessive thoughts reinforce the cycle, creating an ongoing psychological burden, consuming significant time, disrupting routines, impairing cognitive abilities, and hindering social interactions [6,7]. OCD is a leading cause of disability, impacting daily, professional, educational, and social aspects of life. Effective management of OCD is crucial due to the unclear understanding of its pathophysiology. Therefore, treatment failures and recurrences are common for OCD.

OCD is a multi-dimensional neuropsychiatric disorder involving a diverse range of etiological factors such as biological, environmental, genetic, and immunological [7]. Regarding the biology of OCD, alterations in serotonergic and dopaminergic pathways are associated with the pathophysiology and development of the disease as reduced concentrations of monoamines in synapses or altered cortico-striato-thalamo-cortical (CSTC) neurocircuits are frequently observed in OCD patients [6–9]. Besides the altered serotonergic activity, overexcitation of glutamatergic pathways and reduced GABAergic activities are thought to be associated with OCD development [10,11]. Currently, available treatment options for OCD are SSRIs, SNRIs, TCAs, and dopamine antagonists. Besides these pharmacotherapies, cognitive-behavioral therapy is also widely employed as non-pharmacotherapy for the management of OCD. However, among all, 30 to 50% of OCD patients remain nonresponsive to antidepressants or SSRIs/SNRIs therapy [2]. Research reported that SSRIs are not beneficial in 50% of OCD patients, and there was a high recurrence rate following the pharmacological treatment [3], indicating that altered monoaminergic pathways cannot fully explain the pathophysiology of OCD. Therefore, many researchers suggest the involvement of other mechanisms in the pathogenesis of OCD and urge for further research. Recently, immune system alterations or dysfunctions have attracted growing interest among researchers as potential risk predictors or pathophysiological factors for OCD development [12–17]. The prevalence of co-morbidity with OCD is high for several disorders, including the presence of auto-antibodies against neuronal structures like basal ganglia structures [3,4]. The concentrations of pro-inflammatory cytokines in samples of peripheral and CNS were found to be significantly enhanced in OCD patients compared to healthy controls (HCs) [17–24]. OCD patients exhibited 20% to 30% higher neuro-inflammation compared to HCs within CSTC neurocircuits that are involved with OCD symptom development [25]. Cytokine-mediated neuro-inflammation is associated with OCD symptoms in COVID-19 patients, supporting the immune hypothesis of OCD development [26]. Studies reveal higher levels of pro-inflammatory cytokines in OCD patients, such as interleukin-1β (IL-1β), interleukin-6 (IL-6), interleukin-8 (IL-8), and tumor necrosis factor- α (TNF-α) [16]. Research suggests that these cytokines can enter the CNS, activate microglia or astrocytes, and induce neuroinflammatory processes, potentially damaging neurocircuits related to serotonergic or dopaminergic pathways. Also, pro-inflammatory cytokines may decrease synaptic concentrations of serotonin/norepinephrine/dopamine, affecting neurotransmitter availability through various mechanisms [27,28].

Though the dysregulated immune system is involved in the pathophysiology of OCD, there is a controversy regarding the altered levels of these cytokines in OCD patients. Some researchers reported elevated serum levels of pro-inflammatory mediators in OCD patients [29–33]. On the contrary, others either did not find any significant variation in these cytokine levels between OCD patients and HCs or the cytokine levels were found to be reduced significantly [34,35]. These variations in research findings regarding the altered cytokine levels in OCD patients versus HCs might be due to the study design, co-morbidity of other psychiatric disorders, or methodology of cytokine estimation. So, further research is required to address these discrepancies/controversies.

IL-1β, a crucial pro-inflammatory cytokine, regulates innate and adaptive immune responses, with implications in auto-immunity and auto-inflammation [36]. Associated with endogenous pyrogenicity, IL-1β links to the pathophysiology of neurodegenerative and autoimmune disorders, including rheumatoid arthritis [37]. Secreted by monocytes, macrophages, and CNS microglia, IL-1β stimulates the expression of pro-inflammatory mediators and induces IL-6 production by endothelial cells. Clinical and pre-clinical studies reveal the involvement of IL-1β signaling in neuro-inflammatory processes, as evidenced by reduced microglia and astrocyte activation and decreased COX-2 production in IL-1R1 knockout mice [38–40]. Elevated IL-1β levels activate microglia and astrocytes [39,41], leading to the expression of pro-inflammatory cytokines, leucocytic chemokines, leukocytic recruitment to the CNS [42,43], and the overproduction of PGE2 and matrix metalloproteases [38]. IL-1β release from activated microglia contributes to neuroinflammation and induces neuronal death by triggering glial production of neurotoxic substances [37]. Additionally, IL-1β stimulates serotonin reuptake transporter proteins [44], potentially exacerbating OCD symptoms, and enhances glutamate release while down-regulating astrocytic glutamate reuptake, leading to NMDA receptor-mediated excitotoxicity and subsequent neuronal cell death in specific brain regions [45].

IL-6 is another pro-inflammatory cytokine that plays crucial roles in regulating immune and inflammatory responses [46] and thus has been associated with the pathophysiology of several neuropsychiatric and autoimmune disorders. IL-6 is mainly produced and secreted by monocytes and T lymphocytes in the periphery, while astrocytes and microglia are the sources of this cytokine in CNS [47]. The impact of IL-6 on neuro-inflammatory processes in CNS is mainly due to its ability to activate microglia and astrocytes. Several clinical and pre-clinical studies also demonstrated its roles in disrupting neurotransmitter systems, including the alterations of serotonergic, dopaminergic, and glutamatergic pathways.

Emerging evidence suggests the association between immune dysregulation and OCD pathogenesis. However, studies investigating the role of cytokines in OCD are rare [13]. Based on the above background and necessities, the present study aimed to investigate the role of IL-1β and IL-6 in the pathophysiology and development of OCD.

## 2. Material and methods

### 2.1 Study population

We have recruited 58 OCD patients as cases and 30 HCs for this study between September 1, 2023 and October 31, 2023. Patients were enrolled from the psychiatry department of Bangabandhu Sheikh Mujib Medical University (BSMMU), a tertiary care hospital in Dhaka city, Bangladesh. Though we screened 100 patients initially, 42 patients declined, and 58 patients accepted to participate in the study. Age-sex-matched HCs were recruited from surrounding areas of residence of patients. The patients were diagnosed based on the Diagnostic and Statistical Manual for Mental Health Disorders, 5th edition (DSM-5) criteria. The severity of OCD symptoms was assessed according to the Yale-Brown Obsessive Compulsive Scale (Y-BOCS) score. Diagnosis of patients and evaluation of controls were performed by a qualified psychiatrist. We recruited subjects with age between 18 and 60 years in this study. Exclusion criteria for the enrollment of OCD patients were having Y-BOCS scores less than 8, previous history of liver or kidney failure, co-morbidity with other neuropsychiatric disorders. Alcohol and substance abuse, excessive obesity and presence of infectious diseases were considered as additional exclusion criteria. The participants were screened for concomitant auto-immune diseases including rheumatoid arthritis, ulcerative colitis, asthma etc., and the participants having this co-morbidity were excluded from the study. These strict inclusion and exclusion criteria were maintained to minimize the impact of confounding variables to ensure the highest possible homogeneity of the study population. Socio-demographic characteristics were recorded for both patients and HC groups using a structured pre-designed questionnaire. Before the initiation of interviews and taking blood samples from study subjects, informed written consent was taken from each participant and their primary caregivers. The study was conducted according to the Declaration of Helsinki.

### 2.2 The Yale Brown obsessive-compulsive scale

The Y-BOCS is currently popular in measuring the severity of OCD symptoms [2,48]. According to this scale, the obsessions and compulsions of an individual are evaluated by assigning/rating scores of 1 to 4 based on the individual’s response to 10-item questions (5 items for each domain of obsession and compulsion) regarding i) the time spent on intrusive thoughts, ideas or impulses (obsessions) or repeated behaviors or mental act (compulsions) performed to minimize the discomfort or anxiety/distress associated with obsession, ii) the interference of daily activities, social activities, and working ability caused by such thoughts or behaviors, iii) the distress caused by obsession/compulsion, iv) the magnitude of resistance to and v) the degrees of control over these obsessive thoughts/ compulsive behaviors. The total scores of symptom measurement range from 0 to 40 and divide the OCD symptom severity into four categories such as mild, moderate, severe, and extreme obsessive-compulsive symptoms, with 8–15, 16–23, 24–31, and 32–40 scores, respectively.

### 2.3 Blood sample collections and serum separation

For analysis of serum cytokines levels, we collected 5 ml of blood sample from each study subject. After collecting the blood, we kept the sample standing for one hour at room temperature to aid clotting. The samples were centrifuged at 1000g for 15 minutes to get the serum sample from the whole blood. Then, collected serum samples were stored in the Eppendorf tube at -80°C. We used ELISA kits (Boster Bio, USA) to determine the serum concentration of IL-1β and IL-6.

### 2.4 Estimation of serum IL-1β and IL-6 levels

We followed the guidelines provided by the manufacturer during the entirety of the process involved in analyzing the samples. A 96-well microplate precoated with the anti-human interleukin antibody was utilized, with 100 μl of each sample and standard solution being placed in their respective wells. Subsequently, the plate was sealed. It was then incubated at 37°C for 90 minutes. After removing the cover, the contents of the wells were discarded. 100 μl of the cytokine detection antibody (Biotinylated anti-human IL-1β or anti-human IL-6 antibody) was added to each well, followed by a seal and incubation at 37°C for one hour. Following this, the contents of the wells were drained and subsequently rinsed three times with 300 μl of wash buffer. Then, 100 μl of the avidin-biotin-peroxidase complex was incorporated into each well, followed by sealing and incubation at 37°C for 30 minutes. Each plate was subsequently rinsed five times with 300 μl of wash buffer, following the elimination of the liquids. After that, 90 μl of color developing reagent (TMB) was added to each well followed by incubation in dark place for 30 minutes at room temperature. We then added 100 μl of stop solution to each well and within 20 to 30 minutes of stopping the reaction, the O.D. absorbance for each well was measured at 450 nm using ELISA microplate reader. Subsequently, a standard curve for the concerned cytokine was prepared and using the standard equation the serum concentrations of IL-1β and IL-6 were computed and expressed in pg/ml.

### 2.5 Data presentation and statistical analysis

We used GraphPad Prism (version 8.0.1) and SPSS (version 25) for data analysis. Demographic variables and clinical characteristics were analyzed by descriptive statistics and presented as mean±SEM. For continuous variables that follow a normal distribution, we employed a two-tailed unpaired student t-test to determine the mean difference between OCD patients and HCs. The Chi-square test and Fischers’ exact test were applied to evaluate the statistical significance between the groups for the categorical variables. We used scatter dot plot graphs for visual comparison of analyzed cytokine levels between patients and HCs. Correlation analysis including Pearson correlation was performed to find out the potential association among different parameters. The receiver operating characteristic (ROC) curve analysis was performed to assess the diagnostic value of serum IL-1β or IL-6 levels in discriminating OCD patients from HCs. In all cases, statistical significance was considered when p<0.05.

### 2.6 Ethical consideration

The Research Ethics Committee (REC) of the University of Asia Pacific, Dhaka, Bangladesh, has approved the research protocol (Ref: UAP/REC/2023/203-S). We briefed the objectives of the study to all participants, and obtained their informed written consent before participation. We followed the guidelines stated in the Helsinki Declaration to perform this study.

## 3. Results

### 3.1 Socio-demographic characteristics of the study populations

The socio-demographic profiles of OCD patients and HCs are presented in Table 1. No significant variation was observed between patients and controls for age, sex, BMI, marital status, smoking history, and rural versus urban residence (p>0.05). However, we observed differences between the patients and controls in terms of their education level, economic status, occupation, family history, and previous history of OCD.

**Table 1:** Demographic characteristics of the study population.

Parameters	OCD patients (*n* = 58) Mean ± SEM	Healthy controls (*n* = 30) Mean ± SEM	*p v*alue
**Age in years**	32.14±1.43	30.47±2.05	0.502
18–25	20 (34.48%)	11 (36.67%)	
26–35	21 (36.21%)	11 (36.67%)	
36–45	9 (15.52%)	5 (16.66%)	
46–60	8 (13.79%)	3 (10.00%)	
**Sex**			0.440
Male	34 (58.62%)	15 (50.00%)	
Female	24 (41.38%)	15(50.00%)	
**Marital status**			0.650
Married	25 (43.10%)	11 (36.67%)	
Unmarried	33 (56.90%)	19 (63.33%)	
**BMI (kg/m** ^**2**^ **)**	23.97±0.62	24.01±0.57	0.966
Below 18.5 (CED)	01 (1.72%)	0 (0.00%)	
18.5–25 (normal)	40 (68.97%)	18 (60.00%)	
Above 25 (obese)	17 (29.31%)	12 (40.00%)	
**Education level**			0.021
Illiterate	02 (3.45%)	6(20.00%)	
Primary level	8 (13.79%)	2 (6.67%)	
Secondary level	8 (13.79%)	0 (0.00%)	
Higher Secondary level	13 (22.42%)	9 (30.00%)	
Graduate and above	27 (46.55%)	13 (43.33%)	
**Occupation**			0.038
Housewife	13 (22.41%)	7 (23.33%)	
Service	3 (5.18%)	3 (10.00%)	
Unemployed/pensioner	14 (24.14%)	10 (33.33%)	
Student	6 (10.34%)	8 (26.67%)	
Business	14 (24.14)	1 (3.33%)	
Others	8 (13.79%)	1 (3.34%)	
**Economic status**			0.029
High	6 (10.34%)	10 (33.33%)	
Medium	35 (60.34%)	13 (43.33%)	
Low	17 (29.32%)	7 (23.34%)	
**Smoking history**			0.255
Nonsmoker	51 (87.93%)	29 (96.67%)	
Smoker	7 (12.07%)	1 (3.33%)	
**Residence area**			0.983
Rural	28 (48.27%)	15 (50.00%)	
Urban	30 (51.73%)	15(50.00%)	
**Family history of OCD**			<0.001
Yes	19 (32.76%)	0 (0.00%)	
No	39 (67.24%)	30 (100.00%)	
**Previous history of OCD**			<0.001
Yes	34 (58.62%)	0 (0.00%)	
No	24(41.38%)	30 (100.00%)	

Abbreviations: BMI, body mass index; CED, chronic energy deficiency; OCD, Obsessive compulsive disorder; SEM, standard error mean. Data were analyzed either by unpaired two tailed students t test when variables are normally distributed or by Chi-square (and Fisher’s exact test) for categorical data to determine the level of significance between mean difference between OCD patient’s vs HC groups where p<0.05 is considered to be statistically significant.

### 3.2 Clinical characteristics and laboratory findings of the study population

**Fig 1:**
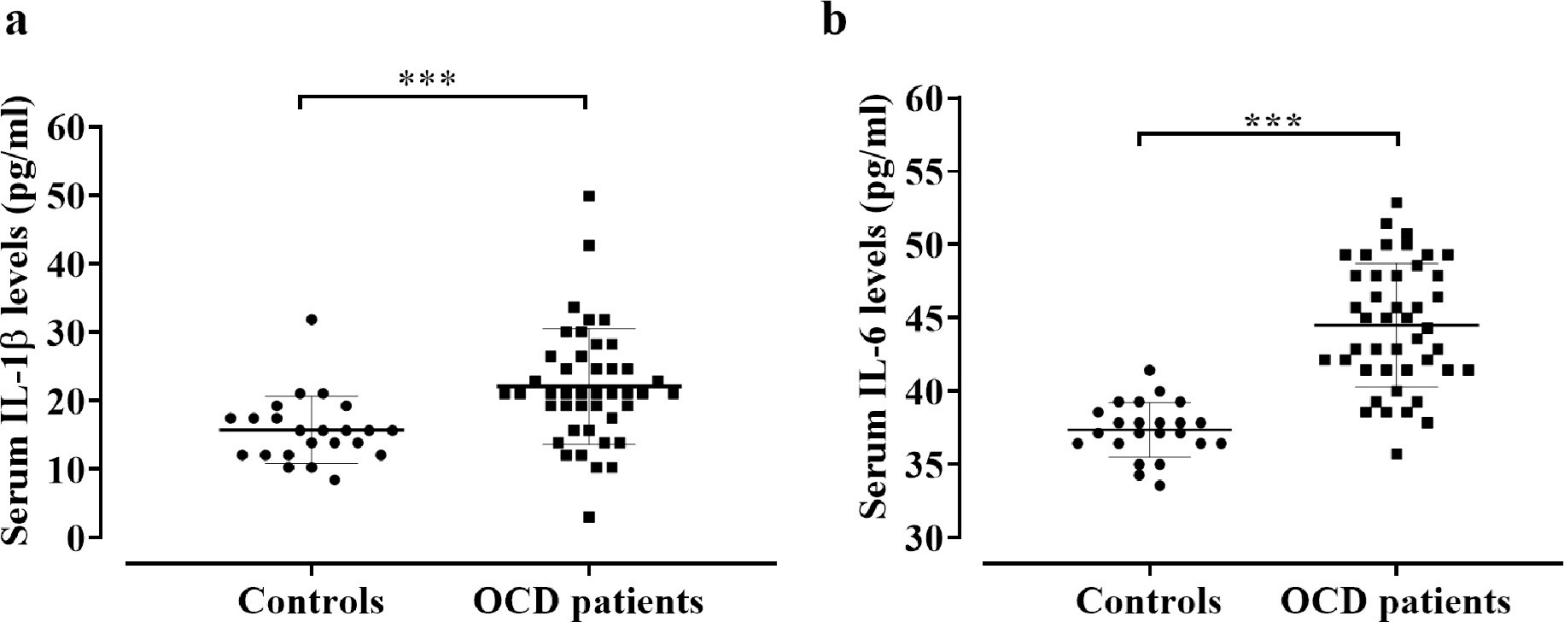
Comparison of serum IL-1β (a) and IL-6 (b) levels between OCD patients and healthy controls groups. where p<0.05 is considered to be statistically significant. ***p<0.001.

We present the clinical profile and laboratory findings of study participants in Table 2. As measured by ELISA assays, OCD patients showed significantly higher levels of serum IL-1β than the HCs (23.68±1.65 pg/ml vs. 15.75±1.02 pg/ml; p = 0.002) (Table 2 and Fig 1). While male OCD patients (25.41±2.30 pg/ml) exhibited significantly (p = 0.006) elevated serum levels of IL-1β compared to male HCs (15.02±0.98 pg/ml), we observed no significant (p = 0.138) variation in IL-1β serum levels between female OCD patients (21.28±2.31 pg/ml) and female HCs (16.43±1.767 pg/ml). We also observed that serum IL-6 levels were significantly higher in OCD patients than HCs (44.97±0.73 pg/ml vs. 37.04±0.35 pg/ml; p<0.001) (Table 2 and Fig 1). Moreover, significantly elevated levels of IL-6 were also maintained for both sexes individually in OCD patients compared to HCs.

**Table 2:** Clinical profile and laboratory findings of the study population.

Parameters	OCD patients (*n* = 58) Mean ± SEM	Healthy controls (*n* = 30) Mean ± SEM	*p v*alue
**Y-BOCS scores**	24.97±0.94	**-**	**-**
**IL-1β (pg/ml)**	23.68±1.65	15.75±1.02	**0.002**
Male	25.41±2.30	15.02±0.98	**0.006**
Female	21.28±2.31	16.43±1.77	0.138
**IL-6 (pg/ml)**	44.97±0.73	37.04±0.35	**<0.001**
Male	45.51±1.14	37.43±0.38	**<0.001**
Female	44.35±0.86	36.69±0.55	**<0.001**

OCD: Obsessive compulsive disorder; Y-BOCS: Yale Brown Obsessive Compulsive Scale for expressing the severity of OCD; SEM: Standard error mean. Two-tailed unpaired student’s *t* test was applied for evaluating statistical significance level between mean difference between cases and controls and p<0.05 was statistically significant.

### 3.3 Association between IL-1β and IL-6 with OCD severity

**Fig 2:**
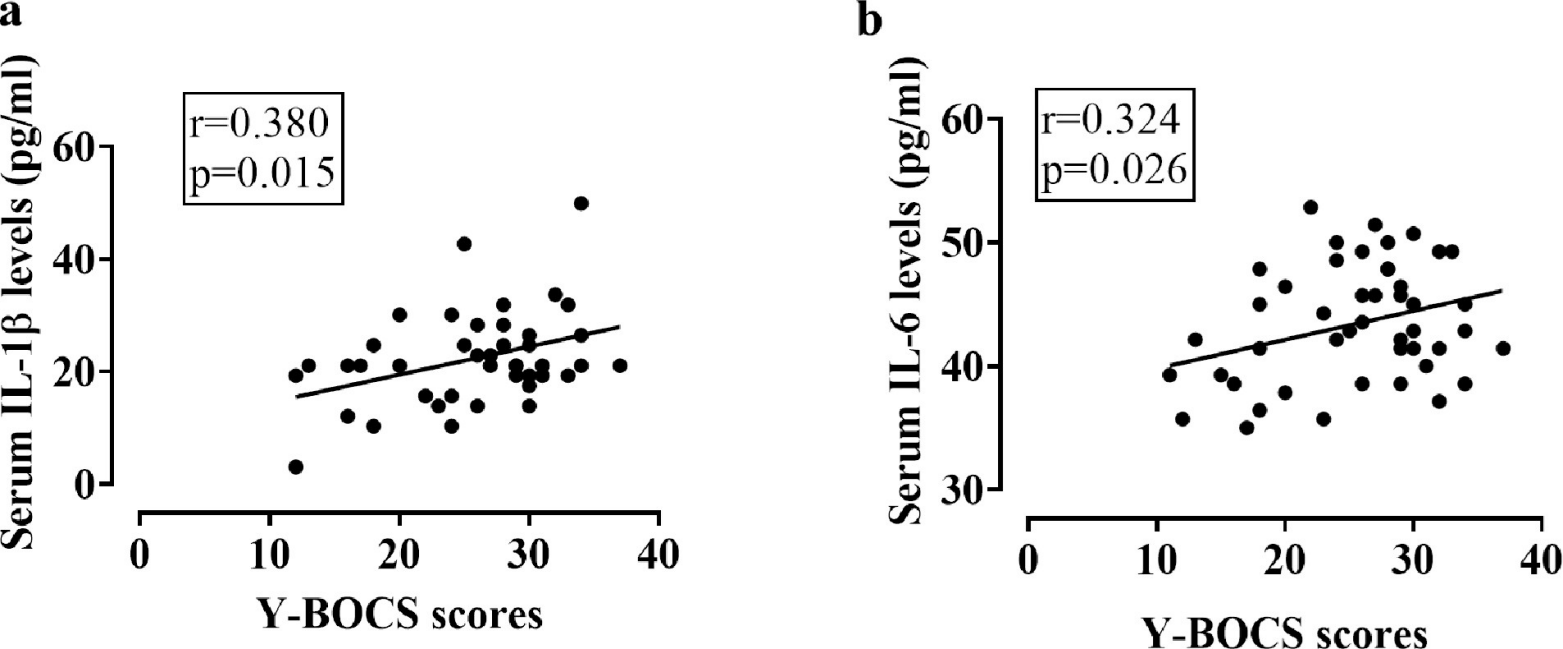
Scatter diagram of Y-BOCS scores vs (a) serum IL-1β levels and (b) serum IL-6 levels in the OCD patients. Pearson correlation analysis was performed to find out the potential association between serum cytokine levels and Y-BOCS scores of OCD. Y-BOCS: Yale-Brown Obsessive Compulsive Scale scores.

We have conducted a correlation analysis to assess the association of elevated serum IL-1β and IL-6 levels with the severity of OCD symptoms as measured by Y-BOCS scores. Analysis showed a significant and positive association between serum IL-1β (r = 0.380, p = 0.015) and IL-6 levels (r = 0.324, p = 0.026) and OCD severity (Fig 2 and Table 3). This correlation analysis also showed that age and BMI were not associated with IL-1β or IL-6 serum levels in OCD patients (p>0.05). On the other hand, while there was no significant correlation between age and Y-BOCS scores of OCD patients, BMI maintained a significant but negative association with Y-BOCS scores (r = -0.275, p = 0.036).

**Table 3:** Pearson correlation analysis among different demographic and clinical parameters in OCD patients.

Correlation parameters	Univariate Pearson’s correlation analysis	
	r	p
Age and IL-1β	-0.206	0.185
Age and IL-6	-0.138	0.364
Age and Y-BOCS	-0.158	0.233
BMI and IL-1β	-0.143	0.359
BMI and IL-6	0.202	0.183
BMI and Y-BOCS	-0.275	**0.036**
IL-1β and Y-BOCS	0.380	**0.015**
IL-6 and Y-BOCS	0.324	**0.026**
IL-1β and IL-6	0.110	0.500

r = Pearson’s correlation co-efficient, p<0.05 is considered to be statistically significant.

### 3.4 Assessment of diagnostic efficacy of serum IL-6 and IL-1β levels by receiver operating characteristics curve analysis

**Fig 3:**
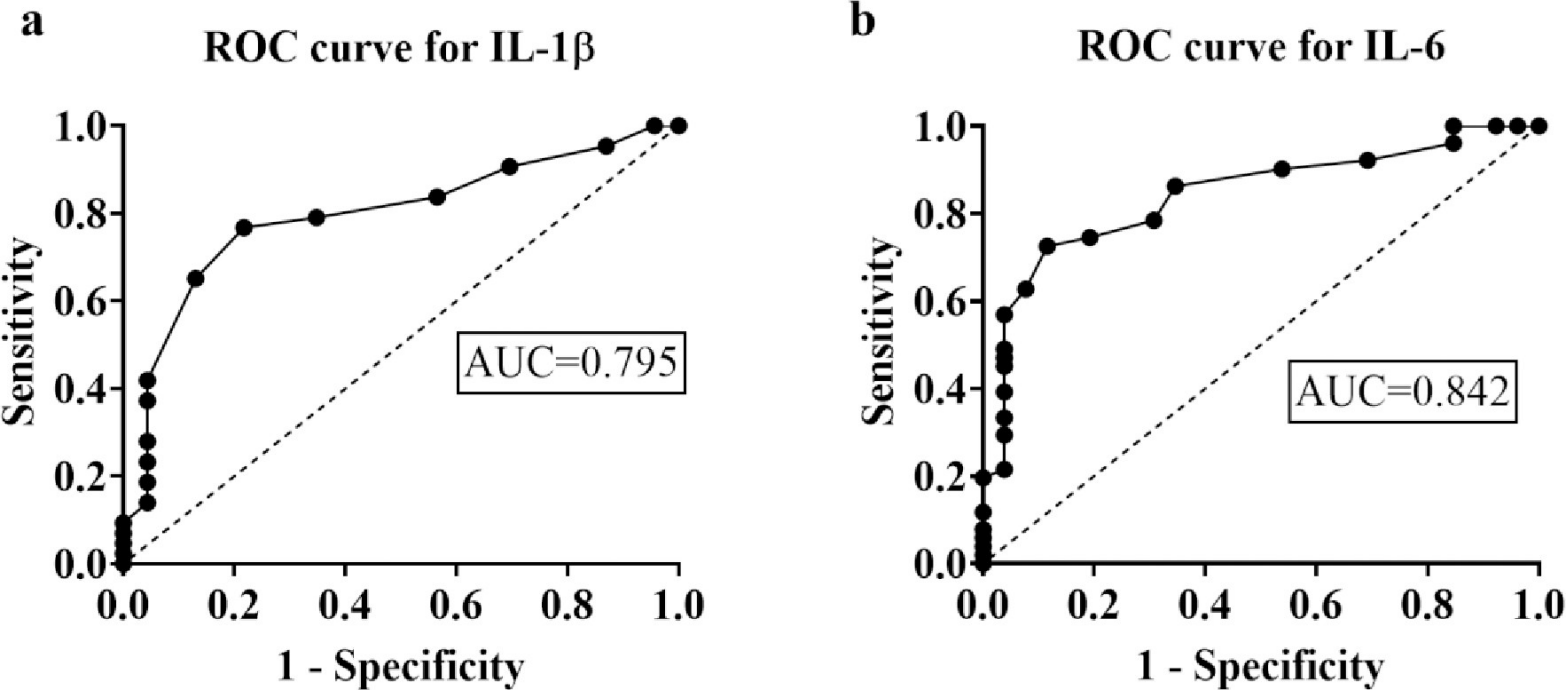
Receiver Operating Characteristic curve for serum IL-1β levels (a) and IL-6 levels (b) in discriminating OCD patients from healthy controls.

ROC curve analysis was performed to examine the diagnostic efficacy of elevated serum IL-1β and IL-6 levels in differentiating OCD patients from HCs (Fig 3 and Table 4). This ROC analysis exhibited that serum IL-1β levels displayed a good predictive performance with an AUC value of 0.796 (95% CI: 0.68 to 0.91) and 76.74% sensitivity and 78.30% specificity at a cut-off value of 18.38 pg/ml. The analysis also showed that compared to IL-1β serum levels, IL-6 levels displayed higher efficacy in discriminating OCD patients from HCs with AUC of 0.842 (p<0.001) and 88.88% sensitivity and 85.71% specificity at a cut-off value of 38.93 pg/ml.

**Table 4:** Receiver operating characteristic curve analysis of serum IL-1β and serum IL-6 levels as discriminators between OCD patients and healthy controls.

Cytokine	Cut-off value (pg/ml)	AUC	95% CI		Sensitivity (%)	Specificity (%)	p value
			Lower bound	Upper bound			
IL-1β	18.38	0.796	0.68	0.91	76.74	78.30	**<0.001**
IL-6	38.93	0.842	0.75	0.93	88.88	85.71	**<0.001**

AUC: Area under the curve; CI: Confidence interval; IL-1β: Interleukin-1β; IL-6: Interleukin-6.

## 4. Discussion

The pathophysiology of OCD is not well understood as the current hypothesis of this disease pathology regarding alteration in the monoaminergic neurotransmitter system is not adequate to explain the pathogenesis of OCD. So, further exploration of the mechanism of pathogenesis is required to unravel the molecular targets for drug development for the disease. Therefore, we conducted this comprehensive case-control study evaluating the association between serum IL-1β and IL-6 levels with OCD symptom severity. We observed a significant elevation in serum IL-1β and IL-6 levels in OCD patients compared to HCs. These results suggested that OCD patients displayed heightened inflammatory responses compared to HCs and that these elevated pro-inflammatory cytokines might be associated with the pathophysiology of OCD. Our results are consistent with those reported by several studies that also observed a significant elevation of IL-1β and or IL-6 serum levels in OCD patients compared to HCs [1,16,31,49]. However, our results are incompatible with some other studies that reported decreased IL-6 serum levels in OCD, or the difference of IL-1β or IL-6 levels between OCD patients and HCs did not approach the statistically significant levels [29,34]. We also observed positive correlations for the elevated levels of both cytokines with the severity of OCD patients. Our study findings resonate with those reported by studies that also observed a significant positive correlation between cytokine levels and OCD symptoms severity [14,31]. However, our results are inconsistent with a few studies that failed to find any significant association between blood cytokine levels (IL-1β, IL-6) and Y-BOCS scores [1,29]. The potential of IL-6 serum level as a risk predictor for OCD development was further potentiated by ROC analysis which indicated that IL-6 serum measurement displayed a very good performance in discriminating OCD patients from HC, implying that serum IL-6 levels might be used as a blood-based diagnostic biomarker for OCD. However, further research is warranted to establish their effectiveness as a diagnostic or prognostic biomarker.

Our study findings have several therapeutic and diagnostic implications. Currently available diagnostic assessment tools for OCD include neuroimaging techniques including positron emission tomography and structured or semi-structured questionnaire or interviews-based Y-BOCS scoring. Though neuroimaging can accurately diagnose OCD, it is costly and cannot be used for screening purposes at the mass level to identify the individuals susceptible to developing OCD symptoms [50]. On the other hand, though Y-BOCS scoring displays an effective diagnosis of OCD and is thus currently used as an assessment tool for OCD, it is based on the patient’s subjective response to 10-item questions and thus results in misdiagnosis or underdiagnosis hampering the treatment and management of OCD. Our study results implicate that IL-6 serum levels might have the potential to act as a blood-based biomarker. As estimation of cytokine serum level is easy to conduct, relatively less invasive, inexpensive, and suitable for multiple screening at different time intervals, using IL-6 might offer an objective diagnostic tool and thus would be useful for accurate and early diagnosis. The early diagnosis or detection will lead to early therapeutic interventions and treatment initiation which ultimately will result in better outcomes in the management of OCD. Overall, cytokine-based early diagnosis might decrease the economic and psychological burden on the individual patient, his family, and society. Blood screening at a mass level for estimation of IL-6 might also help identify individuals having risk for developing OCD and this might guide them to initiate counseling sessions and other nonpharmacological management interventions including noninvasive brain techniques such as transcranial magnetic stimulation or transcranial electric current stimulation. These altogether will ultimately decrease the prevalence and severity of OCD cases and the overall treatment or management costs.

As OCD is considered to be multi-factorial neuropsychiatric disorder, a multi-target treatment strategy including both pharmacological therapies and non-pharmacological options including noninvasive brain techniques (NIBs) should be applied for the effective management of this disease. Recent research indicates the effectiveness of NIBs as complementary therapeutic strategies for the treatment and management of OCD. As such, researchers found that transcranial magnetic stimulation (TMS) and transcranial electric current stimulation (tECS) caused a significant alleviation of OCD symptoms [51]. A meta-analysis conducted by Gao et al., (2022) also found that OCD symptoms were significantly reduced by non-invasive brain stimulation techniques including transcranial magnetic stimulation (TMS) and transcranial direct current stimulation (tDCS) [52]. One of the potential implications of our study findings is that screening the IL-6 levels or probably IL-1β levels might be beneficial in decision making of when to initiate this noninvasive brain stimulation techniques for individuals with risk for developing OCD. The correlation of IL-6 or IL-1β with OCD severity also indicated that IL-6 or IL-1β level estimation might be used in evaluating the effectiveness of these neuromodulation brain stimulation therapies.

Another important implication of our study findings is that unraveling the role of immune dysregulation in OCD can be utilized for targeting the biological pathways for drug development for OCD. This will shed some light on a new understanding of the molecular mechanism of the pathogenesis of OCD and thus might be helpful in the development of new therapeutic interventions, especially personalized and precision medicine. Our study results might be a guide to target IL-6 and probably IL-1β-mediated immune signaling at the cytokine level or cytokine receptor level. Monoclonal antibodies targeting IL-6 (examples: tocilizumab, sarilumab, siltuximab) or targeting IL-1Ra (example: anakinra) can be repurposed for the management of OCD as an adjunctive therapy in combination with either NIBs or SSRIs or SNRIs or other antipsychotics to control or alleviate excessive inflammatory responses mediated by IL-6 or IL-1β. Moreover, the intricate relationship between pro-inflammatory responses and OCD pathophysiology might provide a guide for adopting anti-inflammatory treatment strategies in OCD. As such, anti-inflammatory drugs (NSAIDs) including COX-2 inhibitors such as celecoxib could be used as combination therapy with SSRIs or NIBs in OCD management.

We tried our best to minimize the effect of confounding variables on the research question of potential association between serum cytokine levels and OCD disease severity. We recruited age-sex-matched HCs so that the effect of these variables could be neutralized. We also excluded the patients having chronic or recent infectious diseases so that the impact of infection on cytokine levels can be omitted. We also excluded any patients having co-morbidity of other neuropsychiatric disorders including major depression, anxiety disorder, and panic disorders to remove the impact of depression or anxiety on serum cytokine levels. These strict criteria and study protocols helped us enormously to generate a mostly homogenous study population. To the best of our knowledge, our study results for the first time revealed a significant association between IL-6 and probably IL-1β and OCD symptom severity, underscoring the intricate and complex interplay between pro-inflammatory mediators and OCD pathophysiology.

Our study has some limitations that need to be acknowledged. Firstly, the small sample size is the major limitation of our study. Studies with larger sample cohorts are required to explore the role of dysregulated immune functions in the pathophysiology of OCD. Another limitation of our study is its case-control study design which is correlational and thus unable to establish the causality between elevated serum levels of IL-6 or IL-1β and OCD development or pathophysiology. So, we cannot conclude whether elevated serum cytokine levels are the etiological factors for OCD development or just mere reflections of OCD pathophysiological alterations. Longitudinal studies are required to unravel this dichotomy. Although our study protocol considered some confounding variables and restricted the impact of them on study findings, we did not consider the impact of other co-variates including diet, environmental factors, and genetic factors which might have some effects on serum cytokine levels. Another limitation of our study design is that we have excluded OCD patients having co-morbidity with GAD. As intrusive thoughts, ideas or impulses (obsessions), repeated behaviors, or mental acts (compulsions) are also recognized as methods individuals employ to cope with anxiety, it is quiet challenging to categorize generalized anxiety disorder (GAD) as an exclusion criterion. Despite these, the criteria for OCD and GAD are different in the DSM-V criteria. We followed this DSM-V criteria and a psychiatrist from BSMMU hospital carefully diagnosed and identified the co-morbidity of GAD with OCD and we excluded those patients from the study.

## 5. Conclusion

The present study provides valuable insights into the intricate relationship between dysregulated inflammatory responses and OCD pathophysiology. Increased serum IL-1β and IL-6 levels demonstrated a consistent and significant correlation with the severity of OCD, indicating a potential association with the pathophysiology of the disease. As such, serum IL-1β and IL-6 measurement might offer effective blood-based biomarkers for the risk assessment of OCD. Also, this study contributes to advancing our understanding of immune-mediated mechanisms in OCD, opening avenues for future research and therapeutic developments. We recommend further studies with large and homogeneous samples from different populations to confirm these findings.
